# Supplementary material for: Multi‐dataset identification of innovative feature genes and molecular mechanisms in keratoconus
Source: J Cell Mol Med. 2024 Sep 19;28(18):e70079. doi: 10.1111/jcmm.70079 (PMC11412914; doi:10.1111/jcmm.70079)
Supplement: Supplementary file 2 — Material S2. [file JCMM-28-e70079-s002.zip › 7.GSEA analysis/1.ARL11/KEGG_INSULIN_SIGNALING_PATHWAY.html]

Details for gene set KEGG\_INSULIN\_SIGNALING\_PATHWAY[GSEA]

|  || Dataset | ARL11.group.cls#h\_versus\_l |
| Phenotype | group.cls#h\_versus\_l |
| Upregulated in class | h |
| GeneSet | KEGG\_INSULIN\_SIGNALING\_PATHWAY |
| Enrichment Score (ES) | 0.18083012 |
| Normalized Enrichment Score (NES) | 0.797497 |
| Nominal p-value | 0.68346775 |
| FDR q-value | 1.0 |
| FWER p-Value | 1.0 |
Table: GSEA Results Summary

  

Fig 1: Enrichment plot: KEGG\_INSULIN\_SIGNALING\_PATHWAY      
 Profile of the Running ES Score & Positions of GeneSet Members on the Rank Ordered List

  

| SYMBOL | TITLE | RANK IN GENE LIST | RANK METRIC SCORE | RUNNING ES | CORE ENRICHMENT || 1 | HK3 | na | 938 | 0.397 | 0.0072 | Yes |
| 2 | PYGM | na | 1375 | 0.350 | 0.0248 | Yes |
| 3 | PDE3B | na | 2025 | 0.295 | 0.0315 | Yes |
| 4 | PIK3R3 | na | 2180 | 0.284 | 0.0515 | Yes |
| 5 | PPP1R3B | na | 2860 | 0.247 | 0.0532 | Yes |
| 6 | PIK3CG | na | 2870 | 0.247 | 0.0743 | Yes |
| 7 | CALML6 | na | 3001 | 0.240 | 0.0912 | Yes |
| 8 | CBLB | na | 3964 | 0.202 | 0.0809 | Yes |
| 9 | PPARGC1A | na | 4007 | 0.200 | 0.0970 | Yes |
| 10 | SHC3 | na | 4454 | 0.184 | 0.1000 | Yes |
| 11 | SOCS3 | na | 4502 | 0.183 | 0.1144 | Yes |
| 12 | FBP2 | na | 4838 | 0.171 | 0.1194 | Yes |
| 13 | INPP5D | na | 5042 | 0.165 | 0.1278 | Yes |
| 14 | ACACB | na | 5077 | 0.163 | 0.1409 | Yes |
| 15 | PCK1 | na | 5840 | 0.141 | 0.1311 | Yes |
| 16 | ELK1 | na | 5841 | 0.141 | 0.1432 | Yes |
| 17 | PRKAR1B | na | 5924 | 0.139 | 0.1529 | Yes |
| 18 | PIK3R1 | na | 6079 | 0.135 | 0.1601 | Yes |
| 19 | SOS1 | na | 6194 | 0.132 | 0.1682 | Yes |
| 20 | FASN | na | 6468 | 0.125 | 0.1711 | Yes |
| 21 | GYS2 | na | 7764 | 0.097 | 0.1420 | Yes |
| 22 | FLOT2 | na | 8673 | 0.080 | 0.1227 | Yes |
| 23 | IKBKB | na | 8698 | 0.079 | 0.1289 | Yes |
| 24 | PIK3R2 | na | 8783 | 0.078 | 0.1332 | Yes |
| 25 | MTOR | na | 8802 | 0.078 | 0.1394 | Yes |
| 26 | PPP1R3D | na | 8899 | 0.076 | 0.1432 | Yes |
| 27 | RPTOR | na | 9025 | 0.074 | 0.1460 | Yes |
| 28 | AKT2 | na | 9348 | 0.070 | 0.1427 | Yes |
| 29 | PYGB | na | 9443 | 0.068 | 0.1459 | Yes |
| 30 | INS | na | 9462 | 0.068 | 0.1512 | Yes |
| 31 | PRKX | na | 9610 | 0.066 | 0.1526 | Yes |
| 32 | ACACA | na | 9664 | 0.065 | 0.1567 | Yes |
| 33 | SORBS1 | na | 9757 | 0.063 | 0.1595 | Yes |
| 34 | PHKG1 | na | 9926 | 0.061 | 0.1599 | Yes |
| 35 | SOS2 | na | 9955 | 0.061 | 0.1644 | Yes |
| 36 | INSR | na | 10038 | 0.060 | 0.1672 | Yes |
| 37 | PIK3R5 | na | 10118 | 0.059 | 0.1700 | Yes |
| 38 | RPS6 | na | 10384 | 0.055 | 0.1671 | Yes |
| 39 | RPS6KB2 | na | 10512 | 0.054 | 0.1680 | Yes |
| 40 | BRAF | na | 10628 | 0.052 | 0.1692 | Yes |
| 41 | KRAS | na | 10630 | 0.052 | 0.1737 | Yes |
| 42 | PDPK1 | na | 10673 | 0.052 | 0.1769 | Yes |
| 43 | FOXO1 | na | 10691 | 0.051 | 0.1808 | Yes |
| 44 | MAPK9 | na | 11167 | 0.046 | 0.1710 | No |
| 45 | PRKAA2 | na | 11235 | 0.045 | 0.1730 | No |
| 46 | TSC2 | na | 11315 | 0.044 | 0.1745 | No |
| 47 | GYS1 | na | 11583 | 0.041 | 0.1702 | No |
| 48 | MKNK2 | na | 11748 | 0.039 | 0.1688 | No |
| 49 | FBP1 | na | 12362 | 0.033 | 0.1539 | No |
| 50 | EIF4E2 | na | 12409 | 0.032 | 0.1554 | No |
| 51 | CBL | na | 12670 | 0.030 | 0.1505 | No |
| 52 | PHKB | na | 13081 | 0.026 | 0.1409 | No |
| 53 | MKNK1 | na | 13296 | 0.025 | 0.1368 | No |
| 54 | PIK3CA | na | 13512 | 0.023 | 0.1326 | No |
| 55 | INPP5K | na | 13606 | 0.022 | 0.1318 | No |
| 56 | PPP1CB | na | 13974 | 0.019 | 0.1228 | No |
| 57 | RPS6KB1 | na | 14001 | 0.019 | 0.1237 | No |
| 58 | PRKAR2A | na | 14343 | 0.016 | 0.1152 | No |
| 59 | CALM2 | na | 14606 | 0.014 | 0.1089 | No |
| 60 | PRKAR2B | na | 14661 | 0.014 | 0.1085 | No |
| 61 | LIPE | na | 14686 | 0.014 | 0.1090 | No |
| 62 | PRKCZ | na | 14777 | 0.013 | 0.1075 | No |
| 63 | SREBF1 | na | 14859 | 0.012 | 0.1062 | No |
| 64 | PRKAG2 | na | 15005 | 0.011 | 0.1030 | No |
| 65 | MAPK10 | na | 15299 | 0.009 | 0.0953 | No |
| 66 | PRKCI | na | 16036 | 0.003 | 0.0743 | No |
| 67 | PPP1CA | na | 16505 | 0.000 | 0.0608 | No |
| 68 | PRKAG3 | na | 16733 | -0.002 | 0.0544 | No |
| 69 | MAP2K2 | na | 16997 | -0.003 | 0.0470 | No |
| 70 | PTPRF | na | 17091 | -0.004 | 0.0447 | No |
| 71 | CALML5 | na | 17116 | -0.004 | 0.0443 | No |
| 72 | PHKA1 | na | 17184 | -0.004 | 0.0428 | No |
| 73 | PRKAA1 | na | 17397 | -0.006 | 0.0372 | No |
| 74 | PRKAB2 | na | 17459 | -0.006 | 0.0360 | No |
| 75 | AKT1 | na | 18085 | -0.011 | 0.0188 | No |
| 76 | SOCS4 | na | 18164 | -0.011 | 0.0176 | No |
| 77 | HRAS | na | 18435 | -0.013 | 0.0109 | No |
| 78 | RHEB | na | 18497 | -0.014 | 0.0103 | No |
| 79 | CALML3 | na | 18646 | -0.015 | 0.0073 | No |
| 80 | CALM1 | na | 18762 | -0.015 | 0.0053 | No |
| 81 | PRKACB | na | 18969 | -0.017 | 0.0008 | No |
| 82 | BAD | na | 19342 | -0.019 | -0.0083 | No |
| 83 | PHKA2 | na | 19366 | -0.020 | -0.0073 | No |
| 84 | SH2B2 | na | 19646 | -0.021 | -0.0135 | No |
| 85 | HK1 | na | 19947 | -0.023 | -0.0201 | No |
| 86 | EXOC7 | na | 20003 | -0.024 | -0.0197 | No |
| 87 | CBLC | na | 20116 | -0.024 | -0.0208 | No |
| 88 | PRKAR1A | na | 20169 | -0.025 | -0.0202 | No |
| 89 | PTPN1 | na | 20274 | -0.026 | -0.0210 | No |
| 90 | PYGL | na | 20281 | -0.026 | -0.0189 | No |
| 91 | GSK3B | na | 20728 | -0.029 | -0.0293 | No |
| 92 | FLOT1 | na | 20912 | -0.031 | -0.0319 | No |
| 93 | PPP1R3C | na | 21152 | -0.032 | -0.0360 | No |
| 94 | SHC1 | na | 21475 | -0.035 | -0.0423 | No |
| 95 | PKLR | na | 21593 | -0.036 | -0.0426 | No |
| 96 | PPP1R3A | na | 21722 | -0.037 | -0.0431 | No |
| 97 | PRKAG1 | na | 22360 | -0.044 | -0.0577 | No |
| 98 | IRS4 | na | 22518 | -0.045 | -0.0584 | No |
| 99 | MAPK3 | na | 22614 | -0.046 | -0.0571 | No |
| 100 | PPP1CC | na | 22638 | -0.047 | -0.0538 | No |
| 101 | CALM3 | na | 22971 | -0.050 | -0.0591 | No |
| 102 | GRB2 | na | 23043 | -0.051 | -0.0568 | No |
| 103 | RAF1 | na | 23336 | -0.054 | -0.0606 | No |
| 104 | PRKAB1 | na | 23593 | -0.057 | -0.0630 | No |
| 105 | RHOQ | na | 23656 | -0.058 | -0.0598 | No |
| 106 | PRKACA | na | 23665 | -0.058 | -0.0550 | No |
| 107 | PIK3CB | na | 23818 | -0.060 | -0.0543 | No |
| 108 | EIF4E | na | 23881 | -0.061 | -0.0508 | No |
| 109 | TRIP10 | na | 24319 | -0.067 | -0.0577 | No |
| 110 | MAPK8 | na | 24525 | -0.070 | -0.0576 | No |
| 111 | MAPK1 | na | 24626 | -0.071 | -0.0544 | No |
| 112 | GCK | na | 25358 | -0.081 | -0.0685 | No |
| 113 | CRK | na | 25703 | -0.086 | -0.0711 | No |
| 114 | PRKACG | na | 25752 | -0.087 | -0.0650 | No |
| 115 | PIK3CD | na | 25759 | -0.087 | -0.0577 | No |
| 116 | G6PC2 | na | 25991 | -0.090 | -0.0566 | No |
| 117 | SLC2A4 | na | 26172 | -0.093 | -0.0538 | No |
| 118 | ARAF | na | 26195 | -0.093 | -0.0463 | No |
| 119 | RAPGEF1 | na | 26203 | -0.093 | -0.0385 | No |
| 120 | SOCS1 | na | 26619 | -0.101 | -0.0417 | No |
| 121 | HK2 | na | 26843 | -0.105 | -0.0391 | No |
| 122 | PHKG2 | na | 27440 | -0.118 | -0.0462 | No |
| 123 | AKT3 | na | 27547 | -0.120 | -0.0389 | No |
| 124 | NRAS | na | 27790 | -0.126 | -0.0351 | No |
| 125 | CRKL | na | 27878 | -0.128 | -0.0266 | No |
| 126 | TSC1 | na | 28214 | -0.137 | -0.0244 | No |
| 127 | EIF4EBP1 | na | 28886 | -0.154 | -0.0305 | No |
| 128 | SHC2 | na | 29625 | -0.176 | -0.0366 | No |
| 129 | EIF4E1B | na | 29701 | -0.179 | -0.0234 | No |
| 130 | IRS2 | na | 29852 | -0.183 | -0.0119 | No |
| 131 | PDE3A | na | 30097 | -0.191 | -0.0025 | No |
| 132 | PCK2 | na | 30472 | -0.203 | 0.0042 | No |
| 133 | MAP2K1 | na | 31070 | -0.223 | 0.0062 | No |
| 134 | IRS1 | na | 32036 | -0.261 | 0.0008 | No |
| 135 | SOCS2 | na | 33805 | -0.390 | -0.0166 | No |
| 136 | SHC4 | na | 34410 | -0.501 | 0.0092 | No |
Table: GSEA details [plain text format]

  

Fig 2: KEGG\_INSULIN\_SIGNALING\_PATHWAY      
 Blue-Pink O' Gram in the Space of the Analyzed GeneSet

  

Fig 3: KEGG\_INSULIN\_SIGNALING\_PATHWAY: Random ES distribution      
 Gene set null distribution of ES for **KEGG\_INSULIN\_SIGNALING\_PATHWAY**
